# Experiments on norm focusing and losses in dictator games

**DOI:** 10.3389/fsoc.2022.930976

**Published:** 2022-08-26

**Authors:** Ivo Windrich, Sabrina Kierspel, Thomas Neumann, Roger Berger, Bodo Vogt

**Affiliations:** ^1^Institute of Sociology, University of Leipzig, Leipzig, Germany; ^2^Chair in Empirical Economics, Otto-von-Guericke University Magdeburg, Magdeburg, Germany; ^3^University Department of Neurology, Otto-von-Guericke University Magdeburg, Magdeburg, Germany; ^4^Chair in Health Services Research, School of Life Sciences, University of Siegen, Siegen, Germany; ^5^Chair in Health Economics, Institute of Social Medicine and Health Economics, Otto-von-Guericke University Magdeburg, Magdeburg, Germany; ^6^Center for Behavioral Brain Sciences (CBBS), Magdeburg, Germany

**Keywords:** dictator game, losses, loss aversion, descriptive norms, fairness norms, norm focusing, loss attention

## Abstract

We conducted experiments on norm focusing. The tests were carried out with two versions of dictator games: in one version of the game, the dictator had to allocate a gain of €10, while in the other version, a loss of €−10 needs to be allocated. In a first treatment, we focused subjects on the average giving in similar previous dictator games. The second treatment focused subjects on the behaviour of what a self-interested actor should do. In total, *N* = 550 participants took part in our experiments. We found (1) a significant difference in giving behaviour between gain and loss treatments, with subjects being moderately more self-interested in the loss domain, (2) a significant effect of focusing subjects on the average behaviour of others, but (3) no effect of focusing subjects on the behaviour of self-interested actors.

## Introduction

During the COVID-19 pandemic in 2020 and 2021, many countries used non-pharmaceutical interventions to prevent the spread of the virus (Chin et al., 2021). Thus, several countries passed laws which made it obligatory to wear a mask in many public places, such as public transport, shops and public buildings. From a sociological point of view, this COVID-19 prevention measure constituted the social norm of wearing a mask in many countries. This social norm was known by everyone and also widely obeyed (Boesch, 2021), in part because compliance was controlled by the authorities. Thus, we had a social norm with a very high norm awareness (Popitz, 1980) as well as high conformity.

In spring 2022, when the pandemic slowly decreased and the face mask rule was abrogated, an interesting observation could be made. In most European countries, almost everybody immediately stopped wearing masks. In Germany, however, many people continued to wear masks in public places, such as in shopping malls, or even during walks outdoors. Sociologically, this is a fascinating change in the nature of the norm. As long as the norm was controlled by law, people obeyed it in all countries. When the mechanism of external sanctioning ended, norm obedience also stopped in most European countries and the social norm vanished. In Germany, however, people continued to follow the norm, although there was no longer any external sanctioning mechanism. Hence, the social norm transformed into an empirical norm [in the sense of Bicchieri (2006), see below], like a fashion or a custom.

This anecdotal evidence is an illustrative example of a fundamental principle in human behaviour, namely that people tend to do what others do. When you see everyone else stop wearing masks, you will also stop doing so. But, when you observe that many other people continue to wear a mask, you might also continue to do so, despite it no longer being mandatory.

These phenomena can be analysed from a norm theoretical perspective. Specifically, we used the model developed by Bicchieri (2006, 2017), which distinguishes in particular between empirical norms that are solely based on imitation and injunctive norms that ought to be followed. We tested experimentally whether focusing on these two norms enhances norm abidance. As the example of the COVID-19 pandemic shows, in real life many decisions are taken in the loss domain where there is nothing to gain but only more or less to lose. The pandemic is a situation of loss for the society as a whole, where mask wearing is a social norm of fairness to reduce the loss. Therefore, we also tested the model of norm focusing in the loss domain. For the latter, in particular, as there exists little empirical evidence, we confined our test situation to simple decisions with unambiguous norms. Thus, we conducted anonymous one-shot dictator game experiments (Kahneman et al., 1986), which are easy for the participants to understand. In addition, the number of confounding variables is low because dictator games are non-strategic. Furthermore, with this decision to distribute a ‘pie', the universally known social norm of fairness (Boesch and Berger, 2019) is involved. We use sociological norm theory, especially of Bicchieri (2006), as theoretical framework. Hardly anything is known for this model in the loss domain. Therefore, especially for the loss domain our experiments are also empirical-explorative.

We used two experimental treatments. In a first treatment, we focused dictators on the average giving from past experiments to examine whether they would start doing what others do and differ from the equal split. In a second treatment, we focused dictators on the solution that fully self-interested actors should choose, in order to make it more socially appropriate to take this decision. Both treatments were tested in both the loss and the gains domain. Thus, our study contributes to norm focusing in negative dictator games in particular.

In the next section, we discuss the theoretical background to norms in the dictator game and present our hypotheses on norm focusing. In addition, we review the literature on fairness in gains vs. loss contexts, which shows that it matters whether a dictator game is played over losses instead of gains. In the third section, we outline our treatment design, how we implemented losses and how we focused on norms. In the fourth section, results are presented. We find an effect on dictator giving if we focus the subjects on the average behaviour in previous experiments. However, we do not find that focusing on self-interested behaviour affects the fairness norm. Across different norm-focus treatments, dictators in the loss domain demand more than dictators in the gains domain.

## Theoretical background and related literature

The idea that people tend to follow what others do is very old. In sociology, it goes back to Tarde (2017 [1890]) who stated that imitation is a fundamental principle in human society. Another famous sociological approach to model human actions on the idea of imitating others' behaviour is the threshold model by Granovetter (1978), which states that whether a collective action takes place or not highly depends on the threshold people have of how many others take part in it. Transferred to social norms, we find this idea in the norm theory of Bicchieri (2006, 2017), who defines norms based on the preference that others follow the norm too.

In sociology the term “norm” is usually defined empirically as a pattern of behaviour which is common in a specific situation (Popitz, 1980). From social psychology, there comes the distinction between descriptive and injunctive norms, which lies in the normative aspect of the norm. A descriptive norm is the norm of *what is*, whereas an injunctive norm is about *what ought* to be (Cialdini et al., 1991). A descriptive norm is a common pattern of behaviour in a given situation. If this pattern of behaviour is normatively expected by others, it becomes an injunctive norm. The term “injunctive norm” is used in social psychology (Cialdini et al., 1991); in a sociological context, we usually say “social norm” (Popitz, 1980; Bicchieri, 2006).

During the pandemic, people followed a social norm of mask wearing because they believed that it was normatively expected to do so and they preferred to follow this expectation. As soon as the mandatory aspect of mask wearing ended in spring 2022, the social norm transformed into a descriptive norm. People in Germany—in contrast to many other Europeans—still showed this behaviour even though it was no longer normatively expected.

We are investigating such changes in norm compliance with experiments using the dictator game. In this situation, the universal social fairness norm (Boesch and Berger, 2019) of how to divide a good is applicable. Furthermore, a dictator game experiment is a very elementary situation, as no strategic elements are involved. This offers the advantage that changes in dictator behaviour are independent of the receiver and can be ascribed to changes in the experimental environment. Moreover, dictator game experiments are easy to conduct, and good comparability of results across different settings and populations can be achieved (Camerer, 2003).

There is a large stream of economic literature which explains giving in dictator games with preferences for giving (Fisman et al., 2007) or social preferences, such as the inequality aversion (Fehr and Schmidt, 1999; Bolton and Ockenfels, 2000). From our sociological perspective, we explain giving in the dictator game as compliance with a fairness norm (cf. Bicchieri et al., 2021).

### Norms in the dictator game

Therefore, the question arises of which types of behaviour play a role in dictator game experiments. A meta-study by Engel (2011) indicates that there are two fundamental types of giving behaviour, which are the modes of distribution of *N* = 20,813 dictator offers (Engel, 2011; **Figure 2**). The first and most common behaviour is to give nothing to the receiver, this being the solution to the game for purely self-interested dictators who want to maximise their own outcome. We could call this behaviour a descriptive norm, according to Cialdini et al. (1991), as it is the most usual behaviour in that social situation.

The second most common behaviour in dictator games, according to Engel (2011), is the equal split of the “pie”. We assume that this behaviour constitutes fair behaviour and, hence, a social norm of fairness in dictator game experiments. In contrast to the purely self-interested solution of the game, this norm of playing the equal split is an injunctive norm (Cialdini et al., 1991). It is a common pattern of behaviour in a dictator game experiment which has a normative character. Fairness norms and cooperation norms are social norms (Bicchieri, 2006). It is socially approved of by the other participants to split the given “pie” in half, but there is always a temptation to deviate from that social norm and act in a more self-interested manner.

This claim—that there are only two norms in the dictator game—might be seen as a simplification for research purposes. For dictator games with a preceding production phase, several fairness ideals are discussed in the literature (Cappelen et al., 2007). For dictator games with a “manna economy” (Güth and Kliemt, 2003), however, such as those in the experiments analysed here, “it seems rather uncontroversial to assume that people view the fair solution to be an equal distribution” (Cappelen et al., 2007, p. 818). Krupka and Weber (2013) found a way to elicit norms in the dictator game by showing participants the actions of a standard dictator game and letting them rate the social appropriateness of every action on a four-point scale. As a result, the equal split received the highest score of social appropriateness, indicating this to be the social norm in the game. Actions which lead to an outcome for the dictator of at least 70% of the amount attained a negative score of social appropriateness, indicating these actions to be socially disapproved. Altruistic actions whereby the dictator gives more than half of the “pie” to the receiver are also considered socially appropriate (Krupka and Weber, 2013), but those actions occur rather seldom in the game (Engel, 2011). Thus, if we define a social norm as a typical pattern of behaviour which is socially approved, it is legitimate to state that the social norm of fairness in a standard dictator game without a production phase calls on the dictator to divide the “pie” in half. From a norm perspective, the fact that a large number of dictators plays something between the fully self-interested solution and the equal split means that conformity to the fairness norm is usually rather low. This is not surprising since in a fully anonymous dictator game there exists no external sanctioning mechanism.

### Norm focusing in the dictator game

According to the norm theory of Bicchieri (2006), one important condition for people to follow a norm is the expectation that a sufficient number of other people in a given situation will also do so. This is called the “empirical expectation” about the behaviour of others. This idea is similar to Granovetter (1978) threshold model, where ego also follows a certain behaviour as soon as the individual threshold of others already behaving like this is surpassed. Thus, if we tell dictators in a dictator game about the average giving of other dictators (in previous experiments), we change their empirical expectations of what the other dictators will do. Doing so increases the expectation that other dictators will play the average, and, furthermore, it decreases—as soon as the average is below the fairness point—the expectation that other dictators will follow the fairness norm of playing the equal split. Since following a social norm of fairness is conditional on the expectation that others will do likewise, the focus on the average giving behaviour should decrease the conformity with the equal split norm.

The focus on the average giving from past experiments also sets up a descriptive norm of what is usually done in the role of a dictator in a scientific experiment. Recent experiments on the commonness of observed behaviour (Lindström et al., 2018; Bicchieri et al., 2020) have shown that people tend to infer socially approved behaviour from empirical behaviour. Thus, when we tell dictators what others usually do in the same situation, they will conclude that this average giving is the socially approved behaviour. Furthermore, the focus on this socially approved behaviour should make the fully self-interested behaviour less appropriate. Bicchieri and Xiao (2009) showed that empirical expectations of what other people do are better predictions of behaviour than normative expectations of what should be done, if these two expectations contradict each other. The latter is the case if we focus dictators on the average giving. The normative expectation from the fairness norm tells dictators to play the equal split, but the empirical expectation of what is usually done is set by the focus on average giving. Following Bicchieri and Xiao (2009), the latter should have a greater impact on decisions than the fairness norm. Focusing the subjects on past average dictator decisions should concentrate giving behaviour on this point.

Our first norm-focus treatment is therefore called “Average behaviour” and the corresponding hypothesis reads as follows:

(H1) **Concentration of the distribution of a dictator's demands**^1^ **in the “Average behaviour” treatment:** the focus on the average dictator's behaviour in reference experiments will concentrate the distribution of the dictators' demands around this reference value. Thus, we expect a lower variance of the demands in the “Average behaviour” treatment.

In contrast, we want to investigate the effect of a focus on the fully self-interested solution of the dictator game, as this is the most common behaviour (cf. Engel, 2011).

According to Bicchieri (2006), following a social norm is often a rather automatic decision. Bicchieri (2006) distinguishes between two ways of coming to a decision: a deliberational route, which can be seen as a rational choice, and a heuristic route, which means following a predefined script of behaviour without much evaluation of the situation. Following a social norm typically goes along the heuristic route of applying a behavioural rule to a given situation. This idea is highly in accordance with the sociological point of view that social norms are internalised *via* socialisation (Popitz, 1980). Applied to the dictator game, this means that many dictators recognise the dividing task as a situation where an equal split norm is to be executed and, hence, follow a script of splitting the “pie” in half without further evaluation. Our second norm focus comes into play at this point. By focusing dictators on the decision which a self-interested actor should choose, the heuristic route of following a behavioural rule without thinking should be broken and dictators should be pushed to take the deliberational route.

Therefore, with our second treatment, we examine whether focusing on the norm of playing in a fully self-interested manner pushes the decision in the direction of this norm. With this treatment, we particularly target dictators who—without focusing—would have adhered to the fairness norm because dictators that would have chosen a fully or close to self-interested division of the pie anyway cannot be affected by the treatment while those dictators who are actually fair should now deviate from this decision. Thus, our second treatment is called “Self-interested behaviour” and the second hypothesis is:

(H2) **Decrease of fairness in the “Self-interested behaviour” treatment:** the focus on the self-interested solution of maximising one's own outcome increases dictators' demands, on average, and, in particular, the frequency with which completely self-interested demands are made.

### Previous literature on fairness behaviour in gains and loss domain

We want to test our hypothesis not only in a “manna” (Güth and Kliemt, 2003) world where there are only—more or less—gains, but also in a world of losses where there is nothing to gain but only to lose—more or less—as illustrated by the example of mask wearing during the COVID-19 pandemic.^2^ Undoubtedly, adherence to a fairness norm, or cooperation in general, is particularly crucial in a rougher world.

Thus, another research question is whether there is a difference in fairness behaviour between gains and losses and, with respect to norm focusing, whether a given manipulation works in the same way in the gain and loss domains.

The literature on the standard dictator game on gains shows that there is huge evidence of fairness behaviour (Camerer, 2003). A meta-study by Engel (2011) finds that from 328 treatments and 20,813 dictator decisions around 16.7% of the dictators follow the fairness norm exactly by dividing the “pie” in half and nearly two thirds (63.89%) of all dictators give at least some positive amount to the receiver. On average, dictators give 28.35% of the pie (Engel, 2011). A more recent meta-study by Umer et al. (2022) confirms the latter finding and estimates that dictators on average offer 29.65% of the “pie”.

The picture is less clear and comforting in regard to the distribution of losses in the dictator game, where the literature is much scarcer. Although studies vary in their findings about giving in the dictator game, they all have in common that differences between the gains and loss domains are found. Thunström (2019) conducted two dictator game experiments in the gains and loss domains. In the first dictator game experiment, dictators are more generous in the loss domain than in the gains domain. The difference between the first and second experiment is the focus of the players' attention on the payoff. The difference between the preferences for fairness between the gains and loss domains narrows, but the dictators remain less generous in the loss domain. Yin et al. (2017) and Cochard et al. (2020) found similar results, namely that dictators are more generous in the loss domain than in the gains domain. Other studies, however, have found the opposite, namely that dictators are less generous in the loss domain than in the gains domain (Boun My et al., 2018; Fiedler and Hillenbrand, 2020). A look at the literature on the related ultimatum game yields the same picture (Buchan et al., 2005; Lusk and Hudson, 2010; Zhou and Wu, 2011; Baquero et al., 2013). Theoretically, we do not deduce different predictions for the loss and gains domains from the above presented model. However, the empirical evidence suggests that behaviour differs between gains and loss. We will examine some theoretical interpretations of our findings in the discussion. Hence, our third—rather explorative—hypothesis is:

(H3) **Different fairness behaviour in dictator game on losses**: we expect different behaviour in dictator games on losses and on gains.

## Methods and treatment overview

This study uses data from two experiments. The first is the reference experiment reported in Neumann et al. (2018). In the second experiment, conducted in 2019, we generated the data for the norm-focus treatments.

### Game and treatment design

The experiment consisted of playing a one-shot dictator game. In a dictator game, two players interact: one player, the dictator, receives an endowment and decides how to allocate it between themselves and the other player, that is, the receiver. Our data are based on two different focus treatments and one control condition (see Table 1): all three conditions were implemented in the gains and the loss domains. In each condition, participants played a version of the dictator game. Every subject participated in one condition only.

**Table 1:** Overview of treatments.

**Norm focus**	**Gains domain**	**Loss domain**
Control without norm focus	T1: “Control—Gains” *n* = 100	T2: “Control—Losses” *n* = 110
Average dictator's behaviour in the control treatment	T3: “Average behaviour—Gains” *n* = 84	T4: “Average behaviour—Losses” *n* = 86
Fully self-interested solution	T5: “Self-interested behaviour—Gains” *n* = 90	T6: “Self-interested behaviour—Losses” *n* = 80

In total, *N* = 550 subjects participated in the experiments.

Since we played the dictator games without role uncertainty (i.e., players were randomly assigned to the role of dictator or receiver), we received n/2 dictator decisions per treatment.

Dictators in the gains (loss) domain treatments received an endowment of €10 (–€10) and had to decide how much of the pie they wanted to keep and how much they wanted to pass to the receiver (in 50-cent increments). All participants received a show-up fee of €5.

For reasons of comparability, we analyse the dictators' demands in all the conditions. For the conditions in the gains domain, the dictator's demand is the fraction of the pie that they want to keep for themselves. For the conditions in the loss domain, the dictator's demand corresponds to the fraction of the pie (which is a loss) that the dictator wants to pass to the receiver. Since these dictators' demands are directly comparable, we are able to examine how behaviour in the gains domain differs from that in the loss domain.

#### Implementation of losses

In order to compare the behaviour of subjects in dictator games over losses with that of subjects in games over gains, we needed a mechanism to induce losses. As pointed out by Neumann et al. (2017), the implementation of losses is difficult. For ethical and practical reasons, we were not able to take money from participants; at the same time, we had to avoid selection effects. Therefore, we needed a mechanism that compensates subjects for both their participation and their (possible) loss. A mechanism that proved (1) able to ensure that participants perceived losses to be real and (2) effective in overcoming the house-money effect^3^ (Thaler and Johnson, 1990) is the prepaid mechanism, in which the compensation is paid several days prior to the experiment. This mechanism was first introduced by Rosenboim and Shavit (2012) who found that “subjects who received prior incentives made a greater effort to reduce their possible losses than subjects in the on-the-spot group” (Rosenboim and Shavit, 2012, p. 154).

In accordance with this mechanism,^4^ we split all sessions in the loss domain (T2, T4, T6) into two sub-sessions. During the first sub-session, we asked the participants to fill out a questionnaire. This was a standard Big Five survey which took about 10–15 min.^5^ After the survey, we paid all participants a show-up fee of €5 and an endowment of €10 in cash. Furthermore, they had to sign a receipt to state that they had received the money, that they understood that this payment could decrease during the second sub-session and that they would participate in the second sub-session. They were informed that they had to return the money, reduced by a show-up fee of €3, if they did not participate in the second sub-session. The second sub-session took place 2 weeks later. At this sub-session, a dictator game over a loss of –€10 was played. The money, which they lost during this game, was paid back by the participants in cash after the sub-session.

This treatment design might have some drawbacks. First, participants do not really decide on losses. For ethical and practical reasons, we cannot take participants' own money, which is why we needed to use the prepaid mechanism. However, if they receive the endowment in advance, then, from the perspective of mental accounting theory (Thaler, 1999), the subjects look on the endowment mentally as their own wealth if sufficient time passes between the first and second session. This leads to a shift in the reference point, from which they decide. After 2 weeks, the subjects see the endowment as their own money and, thus, decide on a loss. We used the time period of 2 weeks, as proposed by Rosenboim and Shavit (2012). Second, the prepaid mechanism introduces a difference in design to the gains treatment as there are two sessions instead of one. Nevertheless, we do not believe that introducing a first sub-session in the gains treatment would have an impact on dictators' decisions but, rather, that it would make the experiment too complex to communicate to the subjects. Hence, we decided for reasons of practicability to conduct the experiment in one session in the gains treatment.

Implementing losses is a methodological problem, and we always have to lower our sights. However, using the prepaid mechanism to induce losses is, to our knowledge, the best possible design.

#### “Control” treatments

In the reference experiment, Neumann et al. (2018) compared decisions in the described negative dictator game to decisions in a positive (standard) dictator game. Results from this experiment form our control treatments “Control–Gains” (T1) and “Control–Losses” (T2), where no norm focusing is involved. Hence, the results of these dictator games set the baseline to estimate the effects of norm focusing. Participants in all conditions were recruited from the same subject pools, which makes the results for the “Control” condition and the norm-focusing treatments comparable. The empirical average dictator offers from these experiments are used as reference values in the “Average behaviour” treatments.

#### “Average behaviour” treatments

As described in Game and treatment design, the subjects played either a positive or a negative dictator game. The procedure, software and instruction were the same as in the reference conditions, but a sentence which told participants about the average offer in the prior reference experiments was added at the end of the instructions. This sentence was designed to function as a norm focus and change empirical expectations about the behaviour of the other dictators. Thus, this additional sentence induced a descriptive norm about the typical dictator behaviour.

In our treatment “Average behaviour—Gains” (T3), we added a sentence and disclosed the average dictator values from the “Control—Gains” condition (T1) to the participants. This sentence read as follows: “In a previous year's series of experiments, player 1 kept, on average, €6.44 of the pie”. The value of €6.44 was taken from treatment T1, reported in Neumann et al. (2018).

In the corresponding “Average behaviour—Losses” treatment (T4), we disclosed the average dictator values from the “Control—Losses” condition (T2) to the participants. This sentence read: “In a previous year's series of experiments, player 1 bore, on average, –€3.28 of the loss”. As the value for the average offer, we used the average from treatment T3 reported in Neumann et al. (2018).

#### “Self-interested behaviour” treatments

In the “Self-interested behaviour” treatments, we focused participants on the most common behaviour in dictator games, namely to decide in a fully self-interested manner. Focusing on this behaviour could increase the social acceptability of this descriptive norm, on the one hand, and, on the other, stress the fairness norm of playing an equal split. Thus, the treatment is a test of the robustness of the fairness norm.

We used the same procedure, software and instruction as in the “Control” condition. In the “Self-interested behaviour—Gains” treatment (T5), a norm-focusing sentence was added to the instruction which read: “A rational player 1 who wants to maximise her payoff would keep the whole pie for herself.” The corresponding sentence in the “Self-interested behaviour—Losses” treatment (T6) read: “A rational player 1 who wants to maximise her payoff would allocate the full loss to player 2.”

### Experimental procedure

The experiments were conducted at two laboratories: the Leipzig Experimental Laboratory for Social Science (LEx) and the Magdeburg Experimental Laboratory of Economic Research (MaXLab). The experiments were implemented using z-Tree (Fischbacher, 2007), and participants were recruited using hroot (Bock et al., 2014). In total, 550 subjects were recruited (314 at the University of Leipzig and 236 at the University of Magdeburg). Most of the subjects (477) were students from various fields of studies; 312 subjects were female and 238 male. Participants were invited from the same subject pools of LEx and MaXLab as in the reference experiments (Neumann et al., 2018). Therefore, the subjects of both experiments are highly comparable.

All the participants played the dictator game as a one-shot game, using the direct-response method (without role uncertainty) and with a random matching in a between-subjects design, meaning that each subject was in only one condition. We randomly assigned half of subjects to the role of dictator and the other half to the role of receiver. The wording of the instructions was kept as neutral as possible to avoid framing effects (Dreber et al., 2013).

After the participants have made their decisions in the dictator game, they were asked a question to check whether there was, indeed, a fairness norm which tells dictators how to allocate a given pie in the two versions of the dictator game, i.e., to give half the pie in the gains domain and to bear half of the pie in the loss domain, respectively. The question was taken from Bicchieri and Xiao (2009): “Do you think player 1 should divide the gain (loss) approximately equally with player 2?” Respondents were asked to rate their agreement with the statement on a five-point scale from “do not agree at all” to “totally agree”.

## Results

**Figure 1:**
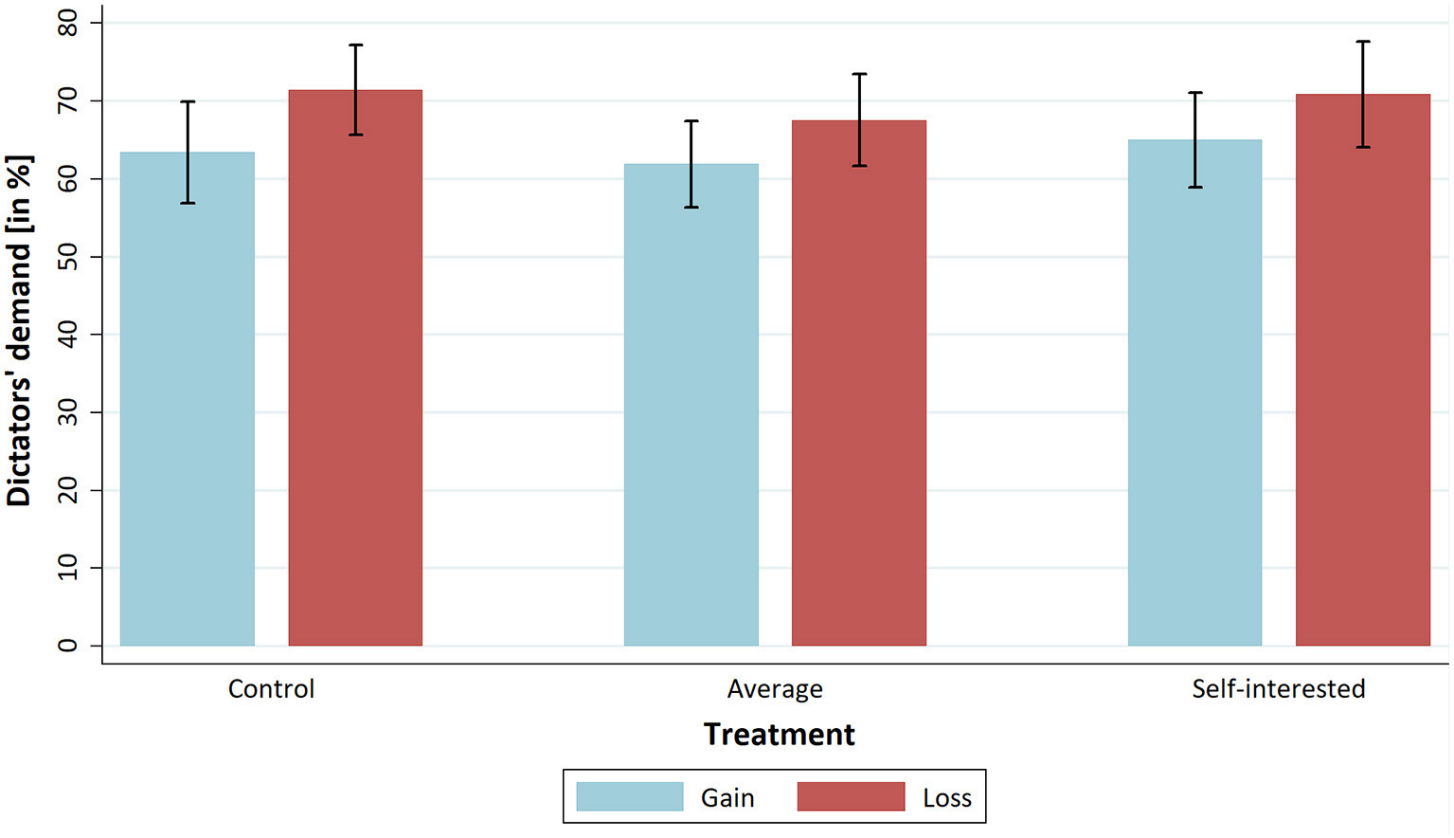
Means of dictators' demands per treatment.

Figure 1 shows the means of the dictators' demands as a percentage of the total pie for the three conditions, separated into gains and losses. In the loss treatment, 100% indicates that the dictator has a loss of zero. In Figure 1, apparent differences in the dictators' demands are visible between gains and loss treatments but not between the “Self-interested behaviour” treatments and the “Control” conditions. Next, we want to present some evidence that a fairness norm existed in the experiment.

### Fairness norm in the dictator game

We wanted to check whether there was a fairness norm in our experiments which tells dictators to play the equal split or something close to it. If we look at the behaviour, overall 41.8% of the dictators played the equal split, with a higher rate of equal splits in the gains domain (48.9%) than with losses (34.8%). This behaviour suggests that there is indeed a fairness norm. However, in the loss domain, this fairness norm is either less present or less adhered to. Theoretically, we expect that it is less frequently adhered to in the loss domain, as investigated in the next section. As another check, we asked about personal fairness beliefs, as in Bicchieri and Xiao (2009). We asked *n* = 340 participants “Do you think player 1 should divide the gain (loss) approximately equally with player 2?” Responses were measured on a five-point scale. The proportion of participants who “somewhat agreed” or “totally agreed” with this equal split norm was 76.4% for gains and 68.7% for losses. Thus, we have a distinct majority of participants who agreed with this norm. However, we also see that the approval is lower in the loss domain. Yet, according to a Wilcoxon rank-sum test on the two distributions, the difference is not significant on the 5% level (*z* = 1.696; *p* = 0.090). However, as this question was asked after the participants had made their decisions, they might have adjusted their answers to the decisions they had already made. Answers to the question and dictators' demands are highly correlated (Spearman rho = −0.75; *p* < 0.001).

### Main effects

Next, we test our hypotheses.

#### Effect of “average behaviour” treatment

**Figure 2:**
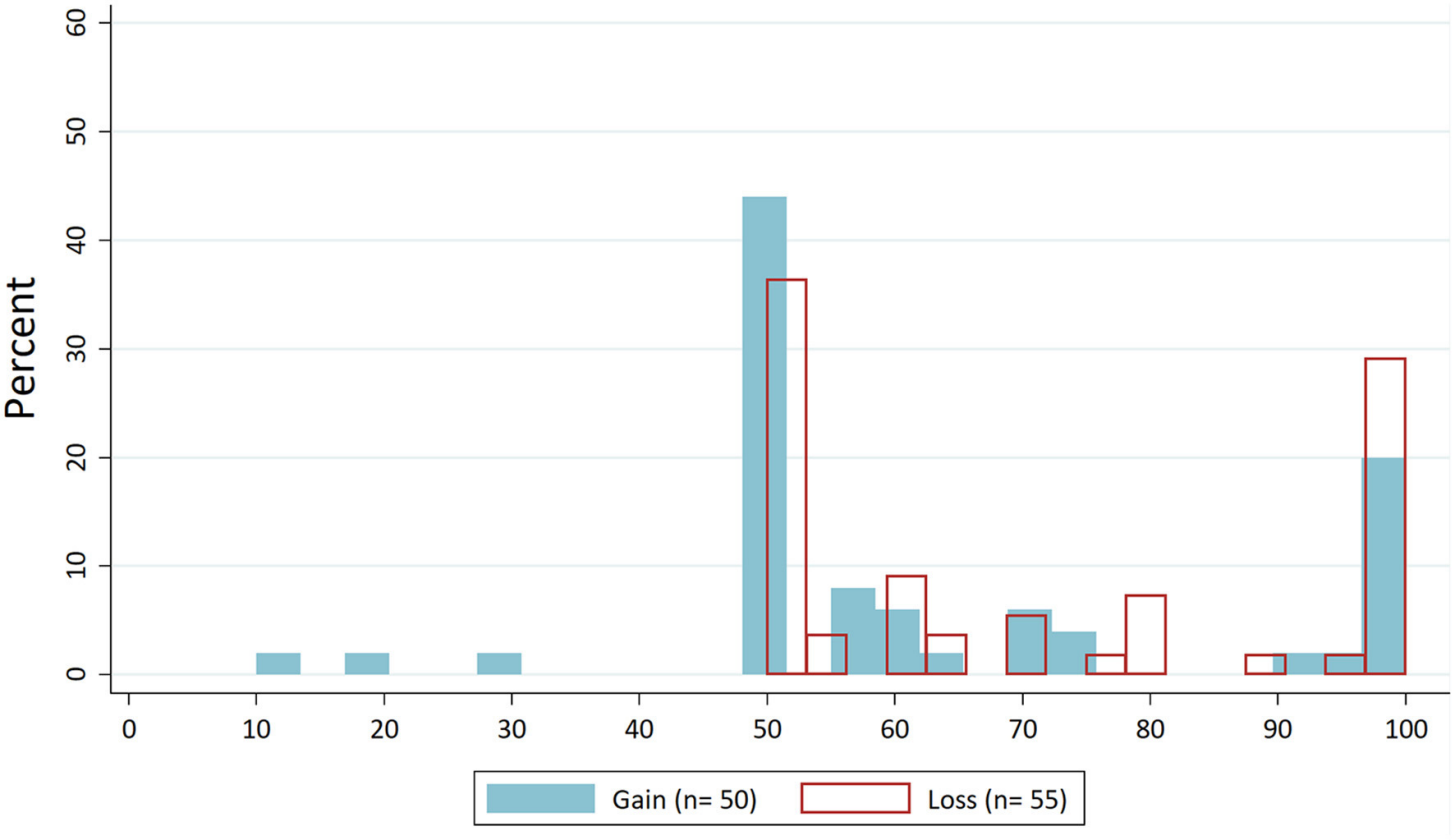
Distribution dictators' demands in “Control”.

**Figure 3:**
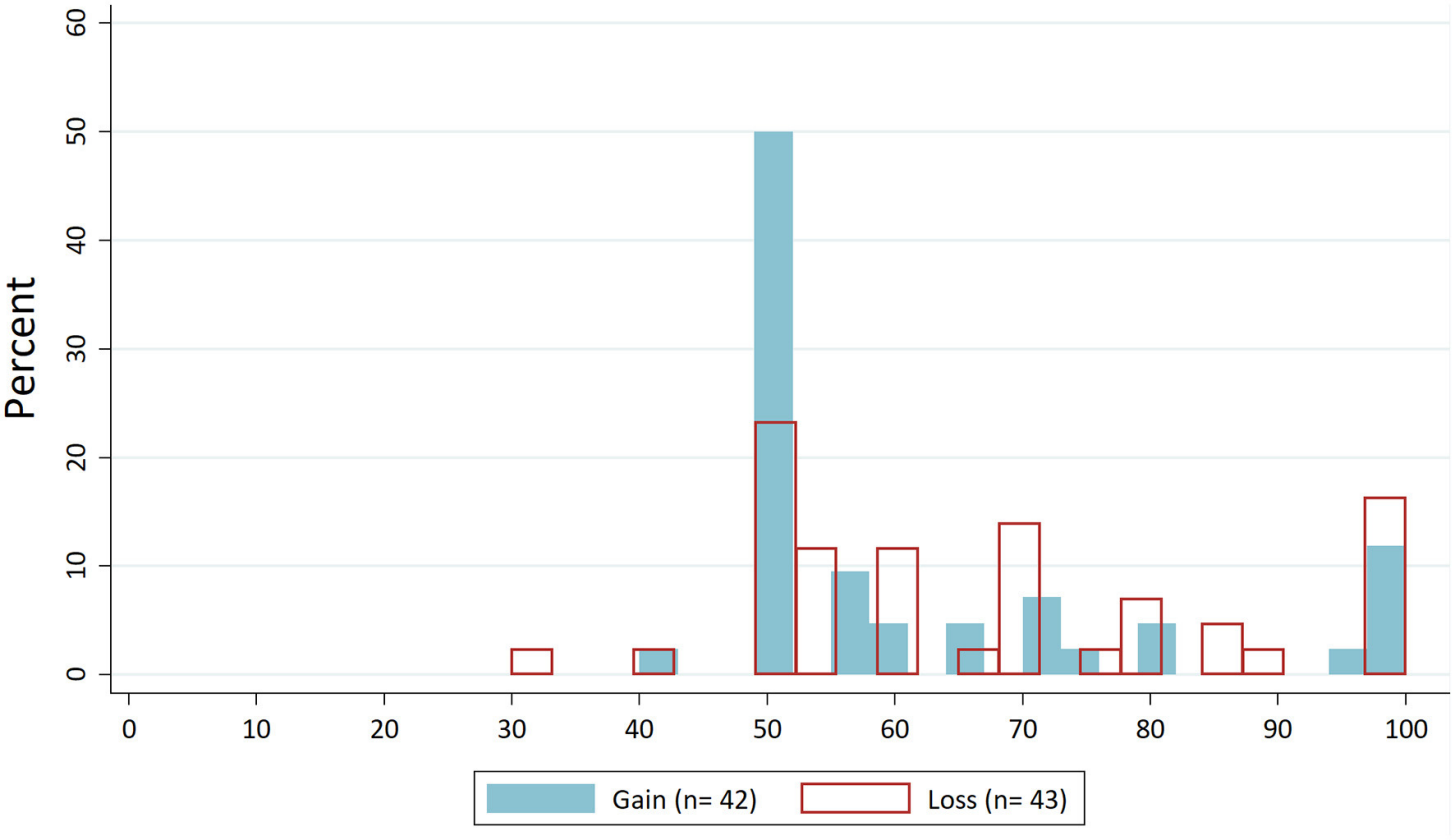
Distribution dictators' demands in “Average behaviour”.

For the “Average behaviour” treatment, we expected that dictators would orient their behaviour around the presented value from control treatments. First, we look at the distributions of dictators' demands. Figure 2 shows the distribution of dictators' demands for the “Control” condition without norm focus. In Figure 3, these distributions are drawn for the “Average behaviour”. If we compare the latter to the distribution for the “Control” condition, we can see, particularly for the loss domain, that the demands are concentrated around the focused value of about €7.

Next, we test the differences in dispersion. In Figure 1, all observations for the four treatments are independent since different subjects participated in each treatment. Hence, for the overall significance of H1, we aggregate the data for gains and losses. The standard deviation of dictators' demands in the “Control” condition was 22.37, and for the “Average behaviour” treatment, it was 18.61. Levene's test shows that this difference is significant (*W* = 6.855, *p* = 0.010). If we additionally test the differences in standard deviations separately for gains and losses, the resulting Levene's test statistics are not significant on the 5% level (for gains: *W* = 3.093; *p* = 0.082; for losses: *W* = 3.511; *p* = 0.064). Thus, H1 is confirmed by the data, but only if data from gains and losses are aggregated. This indicates that the effect of the “Average behaviour” treatment is rather weak.

**Figure 4:**
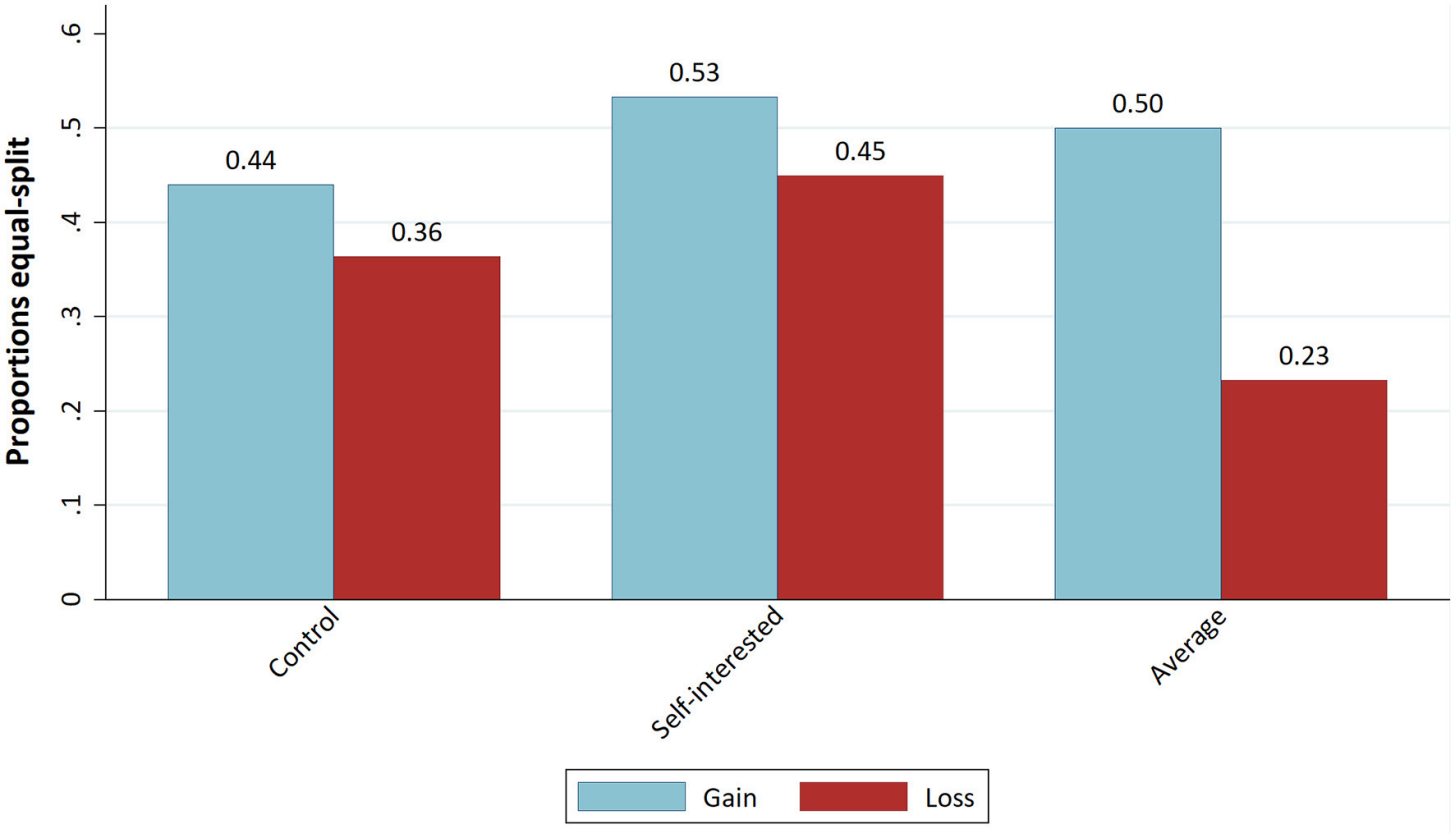
Proportion of equal splits.

**Figure 5:**
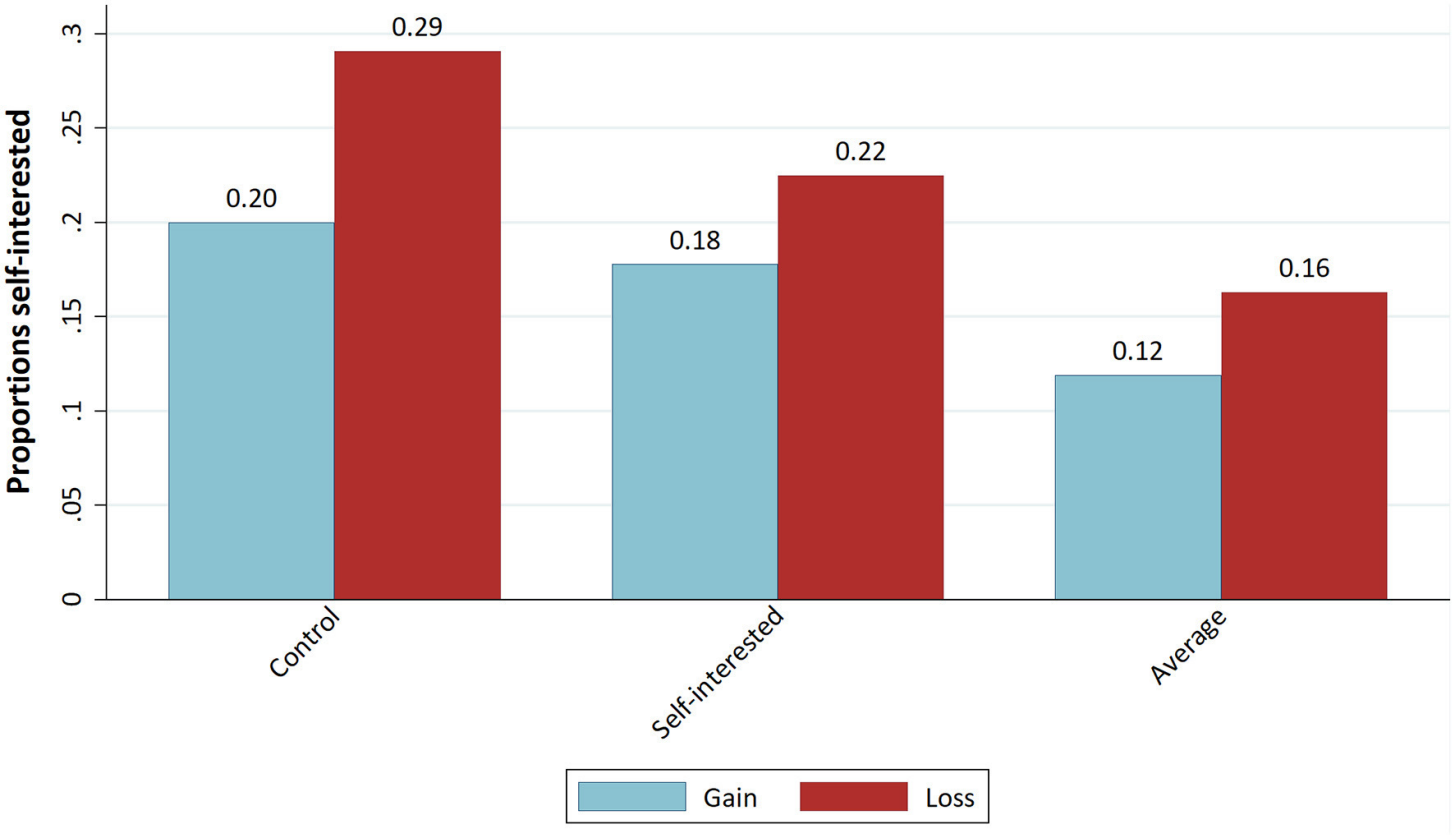
Proportion of fully self-interested decisions.

We further examine the proportions of equal splits per treatment (Figure 4) and fully self-interested demands per treatment (Figure 5). We see a decrease in the frequency of self-interested decisions for the “Average behaviour” treatment in comparison to the “Control” treatment. In the loss domain, moreover, there is a decrease in the frequency of equal splits compared to the “Control” treatment. This finding is therefore in line with H1, though with two-tailed proportion tests it is not significant on the 5% level.

We conclude that H1 is confirmed by the data since the standard deviation of demands decreases for the “Average behaviour” treatment. Dictators concentrate their demands around a presented reference point. Focusing on the descriptive norm of average giving leads to a change in behaviour so dictators tend to follow what others do. Additionally, differences in the proportions of equal splits and self-interested dictators are in the expected direction. Since these were not tested as significant, however, the effect of this norm focusing can only be valued as weak.

#### Effect of “self-interested behaviour” treatment

**Figure 6:**
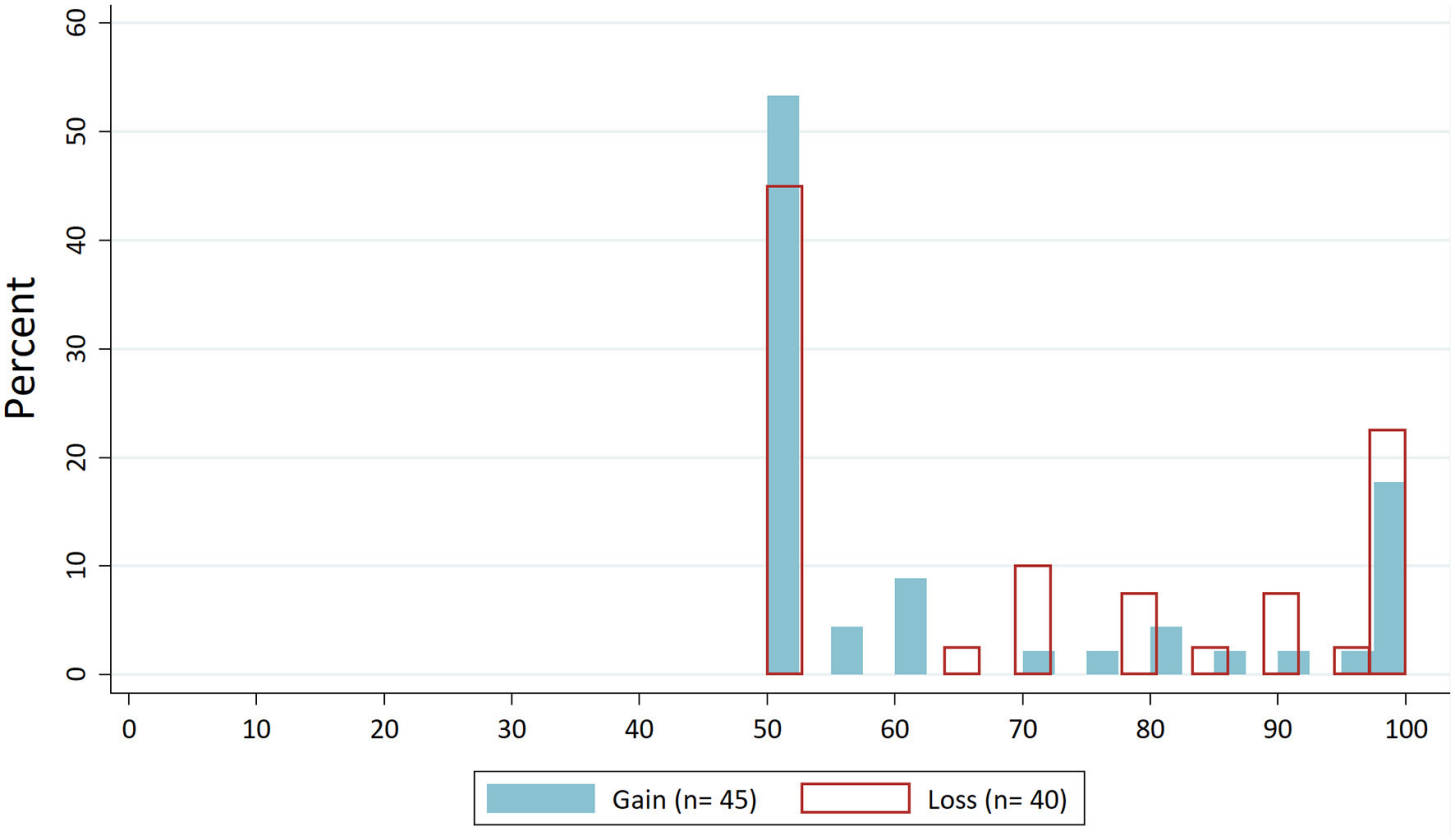
Distribution dictators' demands in “Self-interested behaviour”.

When focusing subjects on the fully self-interested solution, we expected a shift in the distribution of dictators' demands towards this behaviour. Thus, we expected the frequency of self-interested dictator decisions to increase and that of fair decisions to decrease. First, let us look again at the distributions of dictators' demands. If we compare the distributions of the “Self-interested behaviour” treatment (Figure 6) with the “Control” condition (Figure 2), there is no effect on the demands in the gains domain. In the loss domain, there even seems to be a shift in the opposite direction, so that self-interested behaviour decreases and fair behaviour increases.

The latter result is confirmed if we look at the proportions of equal splits (Figure 4) and self-interested decisions (Figure 5). The proportion of equal splits increases in the “Self-interested behaviour” treatment compared to the “Control” condition for both the gains and loss domains, and the proportion of fully self-interested decisions even decreases in the loss domain. However, testing these differences with proportion tests yields no statistically significant difference.

The “Self-interested behaviour” treatment does not show an effect, as expected. In particular, the conformity to the fairness norm of dividing by half remains untouched by our norm focus. This finding contradicts H2. Focusing subjects by giving them analytical information about how to maximise one's own outcome does not affect their behaviour.

#### Effect of loss treatment

In Figure 1, the average demands for the norm-focus treatments and the control condition are presented, grouped by gains and losses, respectively. As predicted by our third hypothesis, there is a difference between dictators' demands. For all three conditions, the average values of dictators' demands are higher in the loss domain than in the gains domain. First, we conduct an overall *t*-test for the difference between gains and losses. In a second step, we test the difference between gains and losses separately for all three conditions. Since we face the problem of multiple hypothesis testing, we use the Bonferroni correction on the significance level. With an overall hypothesis and three partial hypotheses, we adjust the significance level to 0.0125. The overall mean demand for gains is €6.35, while that for losses is €7.01. According to a two-tailed *t*-test, this difference is significant for the corrected significance level (*t* = −2.668; *p* = 0.008). Dictators in the loss domain demanded, on average, 66 cents (around 10%) more than dictators in the gains domain, which, with a Cohen's d of −0.322, is a moderate effect. This finding confirms H3, which states that it makes a difference whether dictator games are played over gains or over losses. If we test the mean with a two-tailed *t*-test for the “Control” condition (*t* = −1.864; *p* = 0.065), “Self-interested behaviour” (*t* = −1.305; *p* = 0.196) and “Average behaviour” (*t* = −1.408; *p* = 0.163) treatments, none of the differences become significant for the Bonferroni-adjusted alpha of 0.0125. The significant difference in mean demands between gains and losses is not found for partial samples of the three conditions.

However, Figure 5 shows that the proportion of self-interested decisions is higher in the loss condition for all norm-focus treatments. Similarly, as shown in Figure 4, the proportion of equal splits is lower in the loss domain for all conditions. Additionally, the decrease of this proportion seems to be particularly strong for the “Average behaviour” treatment. This might indicate an interaction effect of our norm-focus treatment with the loss condition. In Table 2, the frequencies of dictators playing equal split or no equal split are listed for the “Average behaviour” and “Control” treatments, conditional on gains vs. losses treatment. As can be seen, the frequency of equal splits is noticeably lower for the “Average behaviour” treatment in the loss condition. However, a Chi^2^-Test shows no significant difference on the 5% level between gains and losses (Chi^2^ = 7.261; *p* = 0.064).

**Table 2:** Frequencies of equal splits.

	**“Control”**		**“Average behaviour”**		**Sum**
	**No equal split**	**Equal split**	**No equal split**	**Equal split**	
Gains	28 (30.4%)	22 (23.9%)	21 (22.8%)	21 (22.8%)	92 (100%)
Losses	35 (35.7%)	20 (20.4%)	33 (33.7%)	10 (10.2%)	98 (100%)
Sum	63	42	54	31	190

## Discussion and conclusion

We experimentally investigated effects of norm focusing in dictator games on both gains and losses. Theory, the literature and our own data all indicate that being fair and splitting the pie, or bearing half the loss, respectively, is the social norm of what should be done in such a situation and, indeed, the most frequent behaviour.

In our “Average behaviour” treatment, we told dictators the average offer from previous control experiments without norm focusing. By doing so, we focused the participants on the descriptive norm of what is usually done in this situation. From theory (Granovetter, 1978; Bicchieri, 2017; Tarde, 2017 [1890]), as well as from everyday observations such as the norm of mask wearing discussed in the introduction, we know that people tend to follow what others do. From experimental studies Bicchieri and Xiao (2009) and Lindström et al. (2018), we know that empirical expectations of how others will behave in a given situation has an impact on ego's decision. Therefore, we expected dictators' demands to concentrate around the focused average value. This hypothesis was confirmed by our experimental data. Moreover, it could be seen graphically that the proportion of self-interested decisions and, for the loss domain, the proportion of fair equal splits decreased for this treatment, although not in a statistically significant manner. Thus, we conclude that the “Average behaviour” treatment shows at least a weak effect in the expected direction.

In a second norm treatment, we focused participants on the behaviour a self-interested actor would choose in the dictator game. We expected that this would lead to more deliberation and, hence, an increase of deviance from the fairness norm, yet this hypothesis was not confirmed by the data. In fact, the treatment “Self-interested behaviour” even led to some fairer decisions. This might be the case because participants in the simple dictator game fully understand that they can play in either a fair or a self-interested manner. Focusing on self-interested behaviour does not change this situation; indeed, it may even have made the fairness norm salient. Dictators who were already self-interested did not then change their behaviour, but dictators who have an internalised fairness norm might feel even more approved in their conformity when focused on self-interested behaviour.

We found these results of norm focusing for both the gain and the loss domain, but in the loss domain, dictators demanded 66 cents more, overall. In addition, in all experimental conditions, the proportion of self-interested dictators was higher in the negative dictator game with losses than in the positive ones. One possible explanation for this can be deduced from prospect theory (Kahneman and Tversky, 1979; Tversky and Kahneman, 1986), which states that, due to loss aversion, people value losses more than gains. Now, conformity to a social norm of fairness is accompanied by costs. In our case, the material costs are €5 in both the loss and the gains domain. In the gains domain, the €5 are evaluated as a lost gain, or a price of being fair, compared to the reference point of no gain at all. In the loss domain, the costs of fair behaviour are evaluated as a loss which is to be borne compared to the reference point of no loss. As “losses loom larger than gains” (Kahneman and Tversky, 1979, p. 279), it follows that being compliant with the fairness norm in the loss domain is more costly for a dictator, in terms of the value of the monetary costs. This could be one possible explanation of less fair behaviour in the loss domain.

In recent years, several studies have been published which cast doubt on the theory of loss aversion. Yechiam (2019) states that the original studies on loss aversion over-interpreted their findings. Some studies (Gal, 2006; Yechiam and Hochman, 2013b) show that empirical findings, which usually count as evidence for loss aversion, can also be explained by different approaches. There are also studies (Sanders et al., 2021) which fail to replicate the effects of loss aversion.

Since the literature tells us that the existence of a mechanism such as “loss aversion” is doubtful, another psychological approach can be taken to explain different behaviour in a loss context. The loss attention model (Yechiam and Hochman, 2013a,b) states that people have an increased sensitivity to losses, meaning that, if confronted with a decision on losses, people show increased attention. In combination with the decision model developed by Bicchieri (2006), this might be an alternative explanation for higher dictators' demands in the loss domain. Bicchieri (2006) states that there are two forms of decision making: deliberational and heuristic. Following a social norm is usually the heuristic route to making a decision. A dictator notices that the decision is about dividing and remembers a norm of fairness to be applied to this situation. If losses lead to increased attention to the situation (Yechiam and Hochman, 2013b), dictators might switch from the heuristic to the deliberational way of decision making and, thus, find a more “rational choice”. In consequence, loss attention would lead to more deviation from the fairness norm and a more self-interested division of the loss. This would explain higher demands in the loss domain as well as an increase of self-interested decisions in the negative dictator game. If this argument is true, we would also expect an increase in the decision time of the dictator in the loss domain, since attention is considered a “limited resource” (Yechiam and Hochman, 2013b, p. 500). Indeed, we find that dictators in the loss domain needed approximately double the time for their decision making than dictators in the gains domain (27.4 s in the loss domain vs. 14.1 s with gains). This difference is significant (*t* = −4.50; *p* < 0.001). Hence, our data comply with an explanation of increased dictators' demands in the loss domain through a loss attention approach.

Another interpretation addresses an interaction effect of focusing dictators on average behaviour with the loss condition. In regard to imitation, Bicchieri states that in “situations of great uncertainty, it pays to ‘follow the herd”' (Bicchieri, 2017, p. 23). Since a dictator game on losses is accompanied by greater uncertainty than a standard dictator game on a gain, it follows that telling dictators what others did in their situation might have a bigger impact on decisions when the game is played over losses. Indeed, we found for the “Average behaviour” treatment that there was no decrease of equal splits in the gains domain, but there was in the loss domain. However, a statistical test did not yield a significant interaction effect. Nevertheless, we suggest further experimental research on the question whether empirical expectations about the behaviour of others have a stronger impact on behaviour in a loss domain than in the gains domain.

## Data availability statement

The raw data supporting the conclusions of this article will be made available by the authors, without undue reservation.

## Ethics statement

Ethical review and approval was not required for the study on human participants in accordance with the local legislation and institutional requirements. Written informed consent for participation was not required for this study in accordance with the national legislation and the institutional requirements.

## Author contributions

Conception and design: BV, RB, and TN. Collection and analysis of data: IW, SK, and TN. Interpretation of the data and revising the article: IW, SK, TN, RB, and BV. Drafting the article: IW. Funding acquisition: RB and BV. All authors contributed to the article and approved the submitted version.

## Funding

This research was funded by the DFG (German Science Foundation) under grant numbers BE 2373/4-1 and VO 1677/4-1 as part of the research project. The fair division of losses (project number 362787969).

## Conflict of interest

The authors declare that the research was conducted in the absence of any commercial or financial relationships that could be construed as a potential conflict of interest.

## Publisher's note

All claims expressed in this article are solely those of the authors and do not necessarily represent those of their affiliated organizations, or those of the publisher, the editors and the reviewers. Any product that may be evaluated in this article, or claim that may be made by its manufacturer, is not guaranteed or endorsed by the publisher.

## Footnotes

^1^The dictator's demand is the portion which the dictator keeps for themselves; see section Game and treatment design.

^2^Many similar and even more severe real-life examples can be easily imagined for the COVID-19 pandemic and beyond.

^3^This effect is also referred to as “windfall gains” in Arkes et al. (1994) and as “manna economy” in Güth and Kliemt (2003).

^4^The literature provides other methods to implement losses. Berger (2013), for example, used physical activities (frog jumps), while Schosser et al. (2016) used pain to operate losses. Other authors used waiting time as a negative reward (e.g., Berger et al., 2012; Kroll et al., 2014; Doll et al., 2017). The problem with these methods is that there exists no directly comparable gains implementation, as with monetary gains.

^5^The results of this questionnaire are not presented here. In the gains treatment, subjects filled out this survey after the dictator game was played.
