# Supplementary material for: Experiments on norm focusing and losses in dictator games
Source: Front Sociol. 2022 Aug 26;7:930976. doi: 10.3389/fsoc.2022.930976 (PMC9458908; doi:10.3389/fsoc.2022.930976)
Supplement: Supplementary file 1 [file Data_Sheet_1.PDF]

Experiments on norm focusing and losses in dictator games - Appendix

Appendix A. Experimental Instructions – First sub-session loss treatment

Welcome to this experiment and thank you for your participation!

Please be quiet and don't use your mobile phones! Please read these instructions carefully. Communication between the participants is prohibited throughout the experiment. If you have any questions, please raise your hand. An experimenter will then come to your seat and answer your question in private.

This is the first of two appointments of this experiment. Today, you will receive a payment of €25.00. This payment can decrease on the second appointment of the experiment. If anything prevents you from keeping the second appointment, you have to repay the payment minus €3.00 compensation for today's appearance.

Appendix B. Experimental Instructions – Second sub-session loss treatment

General instruction

Welcome to this experiment and thank you for your participation!

Please be quiet and don't use your mobile phones! Please read these instructions carefully. Communication between the participants is prohibited throughout the experiment. If you have any questions, please raise your hand. An experimenter will then come to your seat and answer your question in private.

You received a payment of €25.00 at the first appointment of this experiment. This amount can decrease during today's experiments depending on your decisions and the decisions of other participants. In total, you will play two games over (possible) losses. Afterward, you will be asked to answer a questionnaire.

In the end, we will calculate the total loss you have to repay. This will be done in private. Hence, no other participant will get to know your final payoff.

You and the other participants will make your decisions independently from each other.

The experiment consists of two parts. You will receive separate instructions for each part.

Instruction dictator game

Game description

Two participants in this experiment are randomly selected to play this part together. Player 1 receives a loss of €-10. He has to split the loss between himself and Player 2. Player 1 chooses his own share of the loss (in 50-cent increments) and indicates the corresponding number:

(Screen-shot – Dictator game loss)

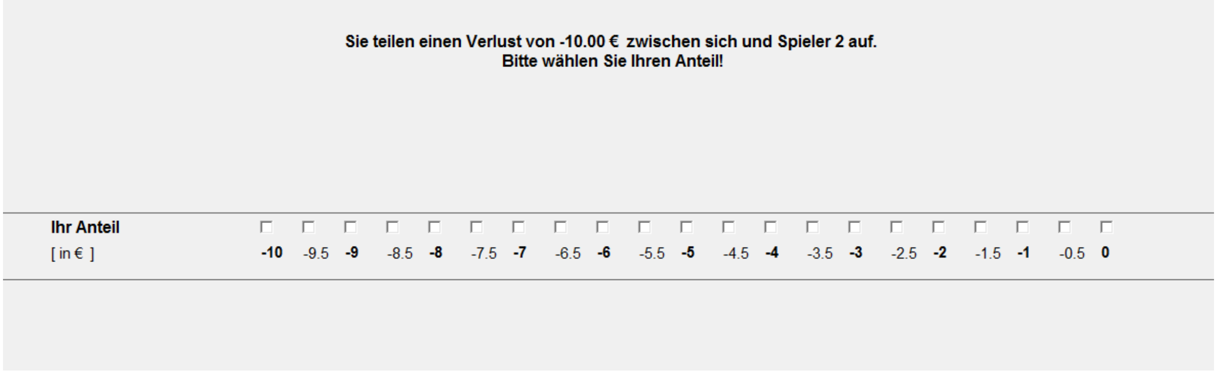

Player 2 receives the difference, thus €-10 less than the share of Player 1. Player 1 decides independently of Player 2, who makes no decision.

'Average behavior' treatment – additional sentence

In a previous year's series of experiments, player 1 bore, on average, -€3.28 of the loss.

'Self-interested behavior' treatment – additional sentence

A rational player 1 who wants to maximize his payoff would allocate the full loss to player 2.

Appendix C. Experimental Instructions – gains treatment

General instruction

Welcome to this experiment and thank you for your participation!

Please be quiet and don't use your mobile phones! Please read these instructions carefully. Communication between the participants is prohibited throughout the experiment. If you have any questions, please raise your hand. An experimenter will then come to your seat and answer your question in private.

You receive a show-up fee of €5 for your participation. Your (final) payoff can increase depending on your and the other participants' decisions. In total, you will play two games. Afterward, you will be asked to answer a questionnaire.

In the end, we will calculate the total gain you will receive. This will be done in private. Hence, no other participant will get to know your final payoff.

You and the other participants will make your decisions independently from each other.

The experiment consists of two parts. You will receive separate instructions for each part.

Instruction dictator game

Game description

Two participants in this experiment are randomly selected to play this part together. Player 1 receives an amount of €10. He has to split this amount between himself and Player 2. Player 1 chooses his own share of the amount (in 50-cent increments) and indicates the corresponding number:

(Screen-shot – Dictator game gain)

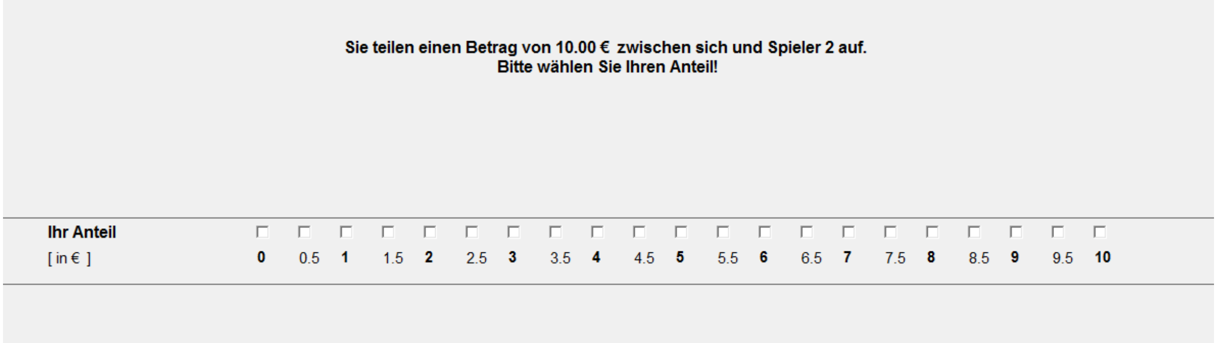

Sie teilen einen Betrag von 10.00 € zwischen sich und Spieler 2 auf.
Bitte wählen Sie Ihren Anteil!

**Ihr Anteil** [in €]	**0**	0.5	**1**	1.5	**2**	2.5	**3**	3.5	**4**	4.5	**5**	5.5	**6**	6.5	**7**	7.5	**8**	8.5	**9**	9.5	**10**

Player 2 receives the difference, thus €10 less than the share of Player 1. Player 1 decides independently of Player 2, who makes no decision.

'Average behavior' treatment – additional sentence

In a previous year's series of experiments, player 1 kept on average €6.44 of the pie.

'Self-interested behavior' treatment – additional sentence

A rational player 1 who wants to maximize his payoff would keep the whole pie to himself.
